# ‘Don’t work more than 3 days straight’: Working conditions as learning conditions in general practice

**DOI:** 10.1016/j.clinme.2026.100588

**Published:** 2026-04-21

**Authors:** Leonard Grant

**Affiliations:** School of Medicine, University of Liverpool, Liverpool, United Kingdom

**Keywords:** Medical education, Curriculum, Working conditions, Workplace learning, Political economy

## Abstract

**Objective:**

To examine how GPs understand their working conditions and to consider what significance this holds for medical education.

**Design:**

Data were collected through online interviews with GPs, conducted between April and July 2023. These data were then subjected to thematic analysis.

**Participants:**

Fifteen GPs were interviewed, including two practice partners, seven salaried GPs, one locum GP and five GPs in training. Participants were recruited through professional networks using purposive sampling.

**Results:**

GPs described intense pressure from increasing demand, large administrative burden and erosion of professional autonomy. Practitioners viewed working full-time as unsustainable, with experienced GPs actively discouraging trainees from taking on such work. The primary coping mechanism that GPs used was reducing their working days, which they considered necessary to continue in practice. The working conditions shape what is understood and transmitted about the profession: that general practice is a fundamentally unsustainable full-time career.

**Conclusion:**

GPs understand their working conditions as untenable and that they must be managed at a personal level. However, these conditions are products of specific political and economic arrangements rather than natural features of medical practice. Medical educators should actively consider what the workplace is teaching and what effects this might have on the future workforce.

## Highlights

• Workplace conditions function as curriculum and teach trainees practice is unsustainable.
• GP trainees learn to protect themselves, primary by reducing working hours.
• Medical education must consider the implications of what the workplace teaches trainees.

## Introduction

### The curriculum in medical education

In medical education, the curriculum is understood not as a static body of knowledge, but as a contested and evolving entity shaped by shifting societal interests and power dynamics.^1^ It is argued that the curriculum produces the appropriate doctors needed for the future,^2^ explicitly setting out to create ʻtomorrow’s doctors’, fit for practice in the 21st century.^3^ As such, curriculum designers must consider what kind of doctors will be produced, and the curriculum is often positioned as the route towards a more socially accountable profession.^4^ The curriculum is primarily conceptualised as a document or set of documents that guide educational practice, comprising structure, content and process.^4^ The dominant view is of the curriculum as a ʻplanned educational experience’ ^5^ that produces specific outcomes, with a linear progression from educational design to action and results.^6^

However, this document-centric understanding risks overlooking the contextual and individual experience of teaching and learning. In medical training – particularly postgraduate training – the workplace is the context of learning. If we accept that curriculum is enacted through experience, not merely designed on paper, then I argue that the workplace and its conditions must themselves be understood as curriculum.

The current conditions of the healthcare workforce in the UK have been described as a growing crisis,^7^ with particular concerns around recruitment and retention of GPs, exacerbated by the recent pandemic.^8^, ^9^ However, if these working conditions are themselves conditions of learning, then this is not only a workforce question, but an educational one.

### The present study

In this study, I examine the working conditions of general practice as experienced by GPs in training and in practice. My aim is not to describe these conditions, but to understand how GPs characterise them and what professional values or behaviours these conditions transmit.

This research seeks to answer the questions:

• How do GPs in training and practice understand and characterise the working conditions of general practice?
• What significance does this have for medical educators?

Although the empirical focus is on general practice, the conceptual argument – that working conditions function as conditions of learning – is intended to resonate across specialties.

## Materials and methods

### Research quality

I consider the principal indication of quality in research to be transparency; all decisions, interpretations and actions related to the research are both articulated and aligned.^10^ Therefore, I aim throughout this paper to render my thinking and reasoning legible. Feminist and liberatory perspectives challenge the notion of research as an objective, value-free activity conducted by a detached researcher,^11^, ^12^ arguing that all research is shaped by the social positions and biases of the researcher. I write in the first person, to keep at the forefront of the reader’s mind that it is with my outlook that I apply various theories and methodologies.

I will briefly discuss my personal context because to interpret research, we must understand the researcher.^13^ I have worked for more than a decade in health professions education, as an academic. I now direct an intercalation programme and a postgraduate clinical education programme. However, I am not a medical practitioner; I come to this research as an outsider, as an observer, and as someone who experiences the curriculum at least one step removed. This transparency extends to my theoretical framework, which shaped every stage of the study.

### Theoretical framework

This study, from research questions to methods of analysis, was guided by the principles of dialectical materialism. Dialectics have existed as long as humanity,^14^ sometimes expressed in the maxim that change is the only constant. Dialectical materialism understands that our consciousness is shaped by our material conditions, that is, the experiences we have in the course of living.^15^ Dialectics orients thinking away from the ‘thing’ and towards ‘the relation’, and from practice to knowledge.^16^ This renders it a suitable framework for this study, which is interested in how experiences in training and practice develop knowledge about the work of general practice.

Consequently, this study does not treat GPs’ accounts as opinions or attitudes, but as expressions shaped by real material conditions – which can then be analysed in terms of their internal tensions, contradictions and relations. Methodologically, this meant tracing how structural conditions appear in individual experience and avoiding explanations that locate problems in individual resilience or failure.

### Methods

To answer my research question, I collected data that contained words, ideas and experiences, as I was interested in how individuals make meaning in practice. I conducted interviews, asking participants about their working conditions, their relationship to work, and how they navigate their practice.

### Participants and recruitment

I identified potential participants as GPs in training or in practice in Britain. Participant recruitment followed a purposive sampling strategy, seeking out interviewees who were most likely to be able to illuminate the issues under study.^17^, ^18^ Data collection does not begin with a blank slate; I had already some ideas about what was going on, from reading and earlier analyses. I aimed to develop a mix of GPs with diversity in years of experience, mode of employment (partner, salaried, locum) and location.

Participants were recruited through personal and professional networks. I contacted 24 potential participants, of whom 15 were interviewed. The participants comprised two GP practice partners, seven salaried GPs, one locum GP and five GPs in training. Eight described their practice as urban, five as rural and two as suburban. Geographically, participants were spread across Britain. Four had undertaken their primary medical qualification outside Britain. Each participant was assigned a random four-character code by which they are referred to throughout this paper.

### Data collection

I interviewed participants online between April and July 2023. Ethical approval was granted by the ethics lead, Faculty of Health and Wellbeing, University of Winchester on 2 April 2023.

An interview schedule was developed in advance, informed by prior reading, but I did not adhere to it rigidly, allowing for flex and flow, spending more time on lines of enquiry that were bearing fruit. I stopped interviewing when I noticed a repetition of ideas and understanding between participants, though I do not invoke data saturation as a concept in this study.

### Analysis

The collection and analysis of data are not meaningfully separable^18^ and I was therefore analysing through the process of data collection. More formally, I followed the thematic analysis approach presented by Braun and Clarke,^19^ which was well adapted to a dialectical analysis, as I was concerned with a ‘philosophy of internal relations’ and in ‘abstracting out the chief patterns’.^14^ I used the qualitative research software ATLAS.ti, without using any of its generative or auto-coding features. Themes were identified based on analytical relevance rather than frequency. I did not carry out any analysis based on demographics or stage of career – this analysis operates at a level of abstraction which is concerned with the totality of the conditions, which arises from my dialectical materialist framework.

To enhance rigour, I kept an audit trail and sought participant validation through member checking,^20^ retaining copies of all correspondence, recordings, transcripts and contemporaneous notes. After each interview I sent the participant a thematic summary for their comment; 12 replied positively and three did not reply.

## Results and discussion

General practices are small- to medium-sized businesses that are commissioned to provide NHS services in a particular area. The challenges and benefits of this model of work I do not explore further here. For the purposes of this study, I will operate at a level of abstraction, looking for general themes and experiences of employment which offer inside into the conditions of work, and consider this as part of what trainees learn.

Please note that I use the term ‘full-time’ to refer to working nine sessions per week. I understand from my participants that each day in practice is very long and amounts to many more hours than would be considered ‘full-time’ in other sectors.

### Mode of employment

The considerations that participants gave towards their chosen mode of employment (partner, salaried, locum) reveal concerns over their working conditions. My sample was disproportionately salaried GPs, partly because my participants were mostly early career GPs (who are more likely to be salaried). Although this may have affected the frequency of quotations, I do not think that it renders the findings invalid.

As a salaried or locum GP, practitioners felt they had fewer responsibilities than partners. Participant 23D8 said:

*They are responsible for management, I am not. It’s their business so they have an interest to make it succeed (23D8 04:05).*

The higher income available to partners did not always offset the added burden of responsibility to both patients and the business. Participant 23D8 was looking to move into partnership, but had reservations about working hours.

However, participants also were aware that being a salaried GP meant having less control over their working conditions and wanted ‘a bit more of a say and input into the care that we deliver, and how we deliver it’ (23AB 02:04). The two locum GPs that I interviewed both commented on how flexible their work could be. However, this was only possible because locum work is ‘much better money than being a salaried GP’ (1126 16:10).

In these motivations for mode of employment, there is a trade-off. GP partners do have more control over their working conditions and generally larger incomes,^21^ but also greater responsibility. Working as a locum may mean flexibility and good remuneration, but it could also mean a lack of job security and the absence of some worker protections.

### The conditions of general practice

I divide this section into two parts. Firstly, I look at the conditions of general practice, how GPs talk about the job and the challenges they face at work. Secondly, I consider the control that GPs can exercise over their conditions.

#### Working conditions

This was a common theme in the interviews, with at least one quotation from every interview appearing in this code.

##### Pressure

Practitioners are under pressure and attempting to manage a perceived or actual demand from patients, exacerbated by government decisions and problems in secondary care. This intense pressure leaves practitioners feeling unable to address problems on a large scale. Participant 0E23, a trainee in an urban practice, explained that ‘if you look at just purely the number of appointments in general practice, that is going up and up and up’ (0E23 29:56).

It is not only the participants that felt this pressure, but also the practice staff. Participant 1702 said that the receptionists would frequently come to their room in tears as they were unable to give needed appointments. Practices responded by putting in place ‘barriers to protect yourself from all the demand’ (065B 44:37).

##### Admin

The participants alluded to GPs facing large admin loads, for which they do not have sufficient dedicated time. They described reading ‘so many, so many blood results’ (1126 37:24). Several participants pointed towards solutions for the increasing admin burden; having dedicated paid admin time (08E9) or employing someone specifically to undertake administrative tasks (181E, 1126).

##### Appointment length

Practitioners felt rushed in their interactions with patients and although the 10-minute appointment caused significant problems for the practitioners, they did not feel able to effect change. The BMA advised that practices move to a 15-min appointment in January 2024^22^ and practitioner conversations around appointment length have been occurring for at least two decades,^23^ with evidence suggesting that longer consultations are associated with a range of positive patient outcomes.^24^

Participants understood that the length of the appointment was a practice decision, but felt that ‘the edicts of the NHS’ (23D8 35:44) determined the appointment length, along with the pressure to see patients (0E23). Practitioner 23D8 thought that increasing the appointment length would lead to reputational damage as fewer patients would be seen in the day.

It is interesting to note that GP partner 25D3 had managed to adjust his practice to both 14 min and double appointments where necessary. The relative autonomy afforded by partnership allowed him to practise medicine in a way that improved both his working conditions and the experience of patients.

##### Increasing complexity

Participants felt that general practice was becoming more complicated. The dominant explanation was not that patients themselves were becoming more complicated, but that ‘medicine is so much more complicated than it used to be’ (23AB 22:09).

##### Lack of autonomy

The participants expressed a lack of autonomy or inability to make decisions about their working conditions. This lack of autonomy leads to worker dissatisfaction, as practitioners feel unable to change their working conditions, see the products of their labour or cooperate fully with other professions.

Practitioners were generally dissatisfied with the expanding role of general practice, feeling that it added unsustainably to their workload and prevented them from giving the care they wanted. Participant 23AB felt that ‘if anything has to be tailed back, it’s non-NHS services that we are essentially delivering for free’ (23AB 33:25). Practitioners also felt that guidelines constrained their professional practice, sometimes to the point of not being able to give treatments which they felt were correct (08E9).

As more decisions are made for GPs outside their workplace, they are less in control of the direction and content of their labour and have no way to alter the trajectory significantly. The participants’ distress over untenable workloads (something that appeared in all of the interviews) and the barriers preventing them from providing adequate care are undoubtedly real.^25^, ^26^

#### Control over conditions

As is to be expected when faced with a difficult working environment, practitioners attempt to exercise control over their conditions. The predominant method among my participants was reducing the number of hours that they worked in general practice.

Practitioners felt that it was impossible to work full-time as a GP. Participant 181E said that it was not ‘sustainable to do nine sessions a week’ (42:59). Participant 0E23 said it was not that practitioners wanted to work part-time, but that they could not with ‘the job as it is at the moment’ (15:43). Participant 25D3 described the ‘domino effect’ of people leaving the system, meaning that those who remain must take on more work (20:17).

However, practitioners would actively discourage attempting to work full-time. Participant 03F0 said ‘I think that’s where madness lies’ (06:48) and that if one of their trainees said that they were thinking about taking on nine sessions, she would discourage them from doing so.

This was a common theme. Participant 2400 also recommended that practitioners do not ‘work more than 3 days straight, definitely don’t work more than 4 days as a GP in a week, you will burn out’ (2400 49:29). Participant 065B explains:

*It’s a funny one because of the way that the GPs naturally protect themselves is to do what I’ve done is develop a portfolio career, but it doesn’t mean I am working less in practice. I see it all the time. (065B 45:43)*

These conditions are also forcing practitioners into the (less autonomous) salaried positions over partnerships. It was generally understood that it is slightly easier as a salaried GP to delineate your working hours, even as participants acknowledged this as detrimental to the profession.

Practitioners control their working conditions primarily by reducing the number of days they work, as the perceived intensity of general practice makes full-time work unsustainable. This reduction in hours is seen as necessary self-protection, but may contribute to workforce imbalance and increased pressure on remaining colleagues.^27^

There was no suggestion made for collective techniques for managing workplace stress; rather, individual methods are favoured. This is isolating and individualising, risking seeing difficult working conditions as a reflection of a doctor’s ability to cope, rather than the conditions themselves being untenable. A study by Whitehead *et al*^28^ found similarly; the GPs that they interviewed experienced burnout as individual feelings of stress, shame, stigma and guilt.

That trainees are being educated into a profession that views itself as unsustainable and under immense pressure must have significant effects on professional identity and long-term commitment to the field. A strong professional identity has been linked to job satisfaction, professionalism and resilience.^29^, ^30^ The pervasive narrative of unsustainability and high levels of job dissatisfaction among established GPs may contribute to a sense of disillusionment and fatalism among trainees. The normalisation of reduced working hours as a necessary coping mechanism may shape trainees’ aspirations for their future careers; rather than seeing general practice as a fulfilling, full-time role, they may be inclined to see part-time working or portfolio careers as the only option for maintaining their wellbeing.

#### Limitations

This study has several limitations. The sample was limited to 15 participants, recruited through professional networks, which may have introduced selection bias. Findings are drawn from interview data alone – the addition of other forms of data may have revealed additional dimensions. Analysis was conducted by a single researcher; I have sought to mitigate this through transparent accounting of my decisions. My position as a non-clinician outsider is a potential limitation, but research has found that GPs interviewing other GPs can make them feel under scrutiny.^31^ The only thing that I can definitely say as to how I was perceived is that some participants assumed I was younger than I am. Finally, the data were collected during a period of NHS industrial tension, and findings may reflect that specific historical moment. However, this dialectical analysis seeks to understand the present, I am not implying that my findings are enduring features of general practice.

### Discussion

Having characterised how GPs understand their working conditions, I now turn to what significance this holds for medical education. GPs respond to adverse working conditions – characterised by increasing lack of autonomy, intense pressure and high admin burden – not by changing the conditions themselves but by reducing their exposure to them, by choosing part-time, salaried or locum positions over partnership. The advice, to not ‘work more than 3 days straight’, is reflective of the normalised wisdom passed from practitioner to trainee – work in general practice is unsustainable and you must protect yourself. Curriculum scholars have identified this ‘unintended’ learning as the hidden curriculum,^32^ but in my work,^33^ I argue that all parts of the curriculum are co-constitutive and so do not draw extensively on the framework of the hidden curriculum, instead considering what it would mean for medical educators to take seriously the working conditions as conditions of learning.

What if, rather than accepting these conditions as inevitable features that must be managed individually, we understood them as products of particular political and economic arrangements? The participants described feeling constrained by ‘the edicts of the NHS’, pressured by demand they cannot control and unable to alter the trajectory of their work. They spoke of profit motives of partners (while this exact situation may not occur in other specialties, there is a large degree of profit generation across the NHS^34^) and of trade-offs between income, control and responsibility. Considering this within the dialectical materialist framework, these are not natural features of medical practice – they are the outcomes of specific decisions about how healthcare labour should be organised and remunerated.

Yet we do not tend, in medical education, to teach trainees to understand their working conditions through the lens of political economy, nor do we consider what they learn from the conditions of work itself. Instead, we teach them to ‘manage the personal and emotional challenges of coping with work and workload’.^35^ What would it mean to take seriously the lessons that trainees learn from their working conditions? If we attend to the workplace as curriculum, we might recognise that we are educating doctors into a particular understanding of their labour: as individuals who must protect themselves from unsustainable demands, who must accept diminished autonomy and reduced hours as the price of survival, and who have no collective recourse to change the structures that constrain them. This recognition could fundamentally alter our approach to both current working conditions and future workforce development. Rather than treating burnout and attrition as largely individual matters, we might understand them as reasonable responses to untenable arrangements. Rather than concentrating on the decision to work less than full-time, we might interrogate what makes this practice impossible. And rather than preparing trainees simply to cope with these realities, we might equip them to name, analyse and collectively challenge the forces that produce them.

## Conclusion

I began from the premise that the workplace functions as curriculum in medical education. Through interviews with GPs in training and in practice, I found that trainees learn that a career in general practice is unsustainable at full capacity, that the work is characterised by increasing pressure and decreasing professional autonomy, and that these features are shaped by external, largely immutable forces. Rather than accepting these conditions as inevitable, I argue that they must be understood as products of specific political and economic arrangements. Medical educators must consider what the workplace is teaching trainees, and what effects this may have on the satisfaction, career intentions and direction of the workforce. This means moving beyond teaching individual resilience, towards helping trainees recognise and respond to the structural conditions that shape their work.

## CRediT authorship contribution statement

**Leonard Grant:** Writing – review & editing, Writing – original draft, Methodology, Investigation, Formal analysis, Conceptualization.

## Ethics approval and consent to participate

Ethical approval for this study was granted on 2 April 2023 by Dr Katherine Cook, ethics lead, Faculty of Health and Wellbeing, University of Winchester. Reference HWB_REC_230201_Grant.

Participants were provided with information sheets and consent forms prior to interview. They provided written consent to participate and were allowed to withdraw up to 7 days after the interview had taken place.

## Funding

This research did not receive any specific grant from funding agencies in the public, commercial or not-for-profit sectors.

## Declaration of competing interest

The author declares that they have no known competing financial interests or personal relationships that could have appeared to influence the work reported in this paper.

## Data Availability

The data that support the findings of this study are available from the corresponding author upon reasonable request.
